# Validation of the parent version of the eating disorder examination-questionnaire adapted for children in parent–child dyads with children with and without loss of control eating

**DOI:** 10.1186/s40337-025-01293-z

**Published:** 2025-06-19

**Authors:** Caroline Lange, Ricarda Schmidt, Anja Hilbert

**Affiliations:** 1 Department of Psychosomatic Medicine and Psychotherapy, Behavioral Medicine Research Unit, Integrated Research and Treatment Center AdiposityDiseases, Leipzig University Medical Center, Leipzig, Germany; 2 German Center for Child and Adolescent Health (DZKJ), Partner site Leipzig/Dresden, Leipzig, Germany

**Keywords:** Eating disorder, Loss of control eating, Binge eating, Validation, Psychometric properties, Assessment, Parent, ChEDE-Q

## Abstract

**Background:**

This study validated a parent-report version of the Eating Disorder Examination-Questionnaire adapted for Children (ChEDE-Q), referred to as the ChEDE-QP, within a population-based sample of parent–child dyads, including both children with and without loss of control (LOC) eating.

**Method:**

The ChEDE-QP was completed by 115 parent–child dyads. Item characteristics, reliability, factor structure, discriminant, convergent, and criterion validity were evaluated. The child version of the Eating Disorder Examination (ChEDE) interview and the ChEDE-Q were used for validation.

**Results:**

Missing item responses occurred in 0–4% of items. Corrected item-subscale correlations were mostly sufficient. Internal consistency ranged from acceptable to excellent. Medium-sized correlations between ChEDE-QP, ChEDE-Q and ChEDE global and subscale scores indicated satisfying convergent validity. Nonsignificant differences between children with and without LOC indicated low discriminant validity. Regarding the presence of LOC eating, parental and child reports were poorly associated.

**Discussion:**

The ChEDE-QP was found to be a reliable questionnaire for assessing children’s eating disorder psychopathology, indicating that parents are helpful for gaining a general understanding of eating disorder psychopathology. However, the agreement on the presence of LOC eating between parents and children was low indicating that the ChEDE-QP should not be used for assessing LOC eating episodes in the absence of child report.

**Supplementary Information:**

The online version contains supplementary material available at 10.1186/s40337-025-01293-z.

## Abstract (plain language summary)

This study tested a new parent-report questionnaire, the Parent Version of the Eating Disorder Examination-Questionnaire adapted for Children (ChEDE-QP). The aim was to help understand children’s eating behaviors. We asked 115 parents to complete the questionnaire, including parents whose children reported loss of control eating and healthy controls. Overall, the ChEDE-QP worked well: it reliably showed whether children had certain eating problems, and it had a high agreement with children’s own responses to questions about eating. However, the ChEDE-QP did not always pick up when a child experienced loss of control eating. This means that while the ChEDE-QP can be useful for getting an overall picture of a child’s eating behavior, parents alone may not always recognize moments when their child loses control of their eating. For that, the child’s own point of view remains essential.

**Supplementary Information:**

The online version contains supplementary material available at 10.1186/s40337-025-01293-z.

## Background

Loss of control (LOC) eating, defined as eating an objectively and/or subjectively large amount of food accompanied by a sense of being unable to stop or control one’s eating, represents a prevalent form of disordered eating among children and adolescents. Metaanalytically, 31.2% of children and adolescents aged 5–21 years report LOC eating [19], i.e., objective (OBEs) and/or subjective binge-eating episodes (SBEs; [14]). Given its cross-sectional and longitudinal association with obesity [6, 38], binge-eating disorder (BED; American Psychiatric Association [APA], [3]; [22, 25, 48], and increased mental impairment [47], LOC eating poses a significant health concern. Consequently, an early detection of children experiencing LOC eating offers a chance for prevention and early intervention, averting the full onset of consequences.

The early identification of eating disorders (EDs) in children relies heavily on reliable information sources [30]. Parents, being close observers of their children’s behaviors, are often recommended as informants, particularly for younger children (≤ 10 years [34],), given that children with EDs might deny their behaviors [40] or lack the cognitive ability for self-reporting, for example, due to incomplete understanding of binge-eating behavior [36]. In a community-based sample of children aged 6–12 years with overweight and normal weight (*N* = 263), 25 children met the criteria for subclinical or full-syndrome BED based on parent report, while child reports identified only 4 children meeting subclinical BED, as assessed by self-report [43]. However, in a community-based sample of adolescents aged 14–16 years (*N* = 7968), adolescents self-reported significantly more OBEs than their parents [45]. In clinical samples, children and adolescents aged 8–18 years endorsing OBEs were more likely to report binge eating than their parents, regardless of whether the Eating Disorder Examination interview (EDE [8, 13, 36]) or self-report questionnaires were used [1]. These findings suggest that parental assessments may serve as a particularly relevant complement to child reports, offering additional insights for improved evaluation. Although the World Health Organization’s World Mental Health Survey indicates that BED typically emerges in late adolescence or early adulthood [28] and is relatively rare in younger populations, with prevalence estimates ranging from 0.7 to 2.3% [47], a systematic review by Byrne et al. [6] highlights that LOC eating is already clinically relevant in middle childhood and that an earlier onset is associated with more severe later psychopathology, including full-syndrome BED, excessive weight gain, and metabolic dysfunction. Notably, previous studies on parent–child agreement in binge eating were based on the Diagnostic and Statistical Manual of Mental Disorders (DSM-IV, DSM-5; APA, [2, 3]) criteria and considered OBEs only. However, the significance of LOC eating is now underscored in the 11th revision of the International Classification of Diseases (ICD-11), which considers recurrent OBEs and/or SBEs as essential for a diagnosis of BED [50]. As a global diagnostic standard, the ICD-11 directly influences treatment decisions for BED, highlighting the clinical importance of assessing the agreement between children and parents on LOC eating. 

Originally developed as a clinical interview in 1987 [7], the Eating Disorder Examination (EDE; [13]) is among the most commonly utilized instruments for assessing EDs and ED psychopathology. In 1994, a questionnaire version, the Eating Disorder Examination-Questionnaire (EDE-Q [12],), was developed to assess ED psychopathology by self-report. Subsequently, adaptations for younger populations, including the child-adapted interview (ChEDE [5],) and its corresponding questionnaire version, the Child Eating Disorder Examination-Questionnaire (ChEDE-Q [17],) emerged. While the ChEDE-Q and EDE-Q have been well-established [4, 24], there is limited evidence for versions designed for parent report. Drury et al. [10] assessed the psychometric properties of a long (28 item) and short (7 items) version of the parent EDE-Q (PEDE-Q) in a clinical sample of 355 individuals aged 12–18 years from the United States and Canada, predominantly diagnosed with anorexia nervosa (AN) and bulimia nervosa (BN, APA, [13]). The PEDE-Q was adapted from the EDE-Q by rephrasing its questions to capture parental observations of the child’s eating behaviors and symptoms. In addition to rewording the original items, guidance was included to help parents identify specific behaviors associated with EDs. For example, item 10 from the EDE-Q, “Have you had a definite fear that you might gain weight?” was modified to “Has your child had a definite fear of gaining weight or becoming fat?” with added prompts to assist parents in recognizing signs like refusal to eat, tantrums, or self-harm threats. For the long PEDE-Q version, the subscales’ (Restraint, Eating Concern, Shape Concern, and Weight Concern) reliability ranged from acceptable to excellent. For convergent validity, the PEDE-Q showed medium- to large-sized correlations (0.41 ≤ *r* ≤ 0.53) and moderate agreement (Intraclass Correlation Coefficient range = 0.58–0.69) with child self-report via the EDE-Q between the corresponding original subscales and global scores. Receiver operating characteristic (ROC) analysis showed that the PEDE-Q had a 34% and 75% chance of identifying full- and sub-syndromal AN, assessed through a semi-structured clinical interview-based on DSM-IV or DSM-5. Also, given a lacking control group without an ED or ED syndrome nothing is known about the PEDE-Q’s discriminant validity, for example, regarding healthy controls. Additionally, the factor structure of the PEDE-Q has not been examined, leaving the underlying structure and the interrelations between items unknown. 

Another parent report questionnaire was developed on the basis of the EDE-Q-Short (EDE-QS) by Gideon et al. [16], comprising 12 items rated on a four-point instead of seven-point Likert scale which are averaged to a total mean score. The EDE-QS-P [49] retained the original items from the EDE-QS but modified the wording to allow parents to respond on behalf of their children (e.g., item 12 “How dissatisfied has your child been with her/his weight or shape?”). The EDE-QS-P was evaluated in 296 treatment-seeking children from the US, aged 6–18 years with diverse, clinician-assessed EDs and their parents, including 14 adolescents with BED. In the study, this prevalence corresponds to only 4.7%, underscoring the low occurrence of BED in younger age groups despite the clinical relevance of LOC eating [6]. In the confirmatory factor analysis (CFA) of the initial 12-item version, item 10 demonstrated poor fit. Since item 10 was used to assess OBEs, its removal left only one remaining item related to binge eating. As a result, the EDE-QS-P’s ability to evaluate binge-eating behavior became limited. However, the 11-item, unidimensional factor model with excellent internal consistency (α = 0.91) revealed a large correlation between the parents’ score on the EDE-QS-P and children’s EDE-Q global score (*r* = 0.82) and medium- to large-sized correlations with child-reported anxiety and depressive symptoms (0.47 ≤ *r* ≤ 0.61). Additionally, parents of children with AN scored significantly higher on the EDE-QS-P global score, thus, perceived more eating disorder psychopathology in their children, compared to parents having a child with avoidant/restrictive food intake disorder (ARFID; APA, [13, 49]) indicating discriminant validity for restrictive EDs. 

In sum, considering the need for reliable parent-based assessments of childhood eating disturbances, preliminary evidence evaluated two adaptations of the EDE-Q or its short form in treatment-seeking children and adolescents with predominately restrictive EDs, leaving questions on the questionnaires’ discriminant and factorial validity, and ability for assessing LOC eating. To address these gaps, this study developed the ChEDE-QP, a parent-report version of the ChEDE-Q to capture parents’ perspectives on their child’s eating behaviors and psychopathology. Unlike the PEDE-Q [10], which is based on the EDE-Q and provides explicit guidelines for answering the questions, the ChEDE-QP uses the simplified wording of the the ChEDE-Q to enhance comparability between parent and child responses. This study examined the ChEDE-QP in 115 parent–child dyads from Germany, with children aged 8–13 years with and without LOC eating. The aim was to evaluate the ChEDE-QP’s psychometric properties, including item characteristics, internal consistency, and factorial, discriminant, criterion, and convergent validity, using interview-based data (ChEDE).

## Methods

### Participants and study design

Data were derived from a study on children’s eating behavior at Marburg University, Germany, focusing on the clinical and experimental characterization of childhood LOC eating (e.g., [9, 23]). Participants were recruited from the community through local schools, where the German version of the ChEDE-Q [20] was administered as a screening tool to identify students with and without reported LOC eating episodes, and utilizing newspaper articles and posters. Ethical approval was granted by the ethics committee of the German Psychological Society. Written informed consent and assent were obtained from parents and children, respectively. Although the recruitment took place in 2005–2007, the study remains relevant several years later considering that well-validated assessment tools were used ChEDE [5, 20, 21], ChEDE-Q [12, 26], the conceptual stability of LOC eating, and its continued clinical significance.

Inclusion criteria for children exhibiting LOC eating (LOC +) required at least one episode of LOC eating within the preceding 3 months, as assessed by the ChEDE [5, 20, 21]. These criteria for LOC-eating frequency and symptom duration were selected in alignment with the subthreshold BED criteria outlined in the DSM-5 [13]. In addition, previous studies have shown that a single LOC episode within the past three months is sufficient to identify higher levels of eating disorder psychopathology, behavioral disturbances, and internalizing problems in those with versus without reported LOC eating [9]. Children in the no LOC eating group (LOC-) required the absence of current or lifetime EDs or disordered eating symptoms (e.g., LOC eating or a history of dieting). Inclusion criteria for both groups included an age range of 8–13 years, and sufficient German language proficiency for the child and the participating parent. To be included in the present analysis, data on the ChEDE-QP had to be available. Exclusion criteria for both groups were compensatory behaviors (more than once over the past 3 months), psychotic disorders in child or parent; medical conditions or medication with an effect on eating behavior or body weight; treatment for overweight; special education; or a planned move or commute time of more than 30 min to the laboratory site. Families were offered a compensation of €100 for full study participation.

### Procedure

After ascertaining inclusion and exclusion criteria via telephone interview, eligible children and one of their parents were invited to a first diagnostic appointment and informed consent and assent were obtained. Diagnostic status was ascertained using the German version of the ChEDE [26], along with objective measurements of height and weight, and the ChEDE-QP. Children in the LOC + group were individually matched with those in the LOC- group based on age, sex, BMI percentile, child’s education (school type and grade), and mother’s years of education. A total of 751 children were contacted for the purpose of this study (schools: 536, advertisements: 215). Following the telephone screening, 624 of these children were excluded (no matching partner: 298; not interested/too far: 143; lack of language skills: 54; no LOC despite positive screening question: 33; compensatory behaviors: 28; sick/in treatment: 32; other reasons: 36), leaving 65 LOC + and 62 LOC- children to participate in the diagnostic session. Following the diagnostic visit, 7 children were excluded (no matching partner: 2; not interested: 2; no LOC: 3), For the present study, 5 children were excluded for this study due to missing data on the ChEDE-QP leaving a total sample of *n* = 115, with *n* = 56 children in the LOC + and *n* = 59 in the LOC- group. A detailed overview of participant recruitment, inclusion, and exclusion is provided in the supplementary flow chart [Supplementary material 1].

### Measures

#### Eating disorder examination adapted for children

The ChEDE [5, 20, 21] is an age-adapted version of the semi-structured expert interview EDE [13, 26] for ED diagnosis and assessment of the specific psychopathology of EDs in children aged 8 and 14 years based on the four subscales: Restraint (RS), Eating Concern (EC), Weight Concern (WC), and Shape Concern (SC). The 35 items, 22 of which were assigned to subscales, referred to the past 28 days and assessed frequencies or intensities on seven-point rating scales (0 = *characteristic was not present*, 6 = *characteristic was present every day/in extreme form*), while 13 diagnostic items referred to the past 3 months for diagnosis of BED, AN, and BN according to DSM-5 [13]. In addition to subscale mean scores, a global mean score was calculated depicting children’s eating disorder psychopathology in general. Evidence on the psychometric quality of the German version of the ChEDE has been provided [22, 25].

#### Eating disorder examination-questionnaire adapted for children

The ChEDE-Q is the child version of the EDE-Q [12, 26], assessing the specific psychopathology of EDs in children aged 8–14 years. Like the EDE-Q, the ChEDE-Q is comprised of 28 items, with 22 items building its four subscales, provided with the same rating scheme as those of the ChEDE. Further 6 items assess diagnostically relevant core behaviors of EDs over the past 28 days, for example, OBEs. Evidence on the psychometric quality of the German version of the ChEDE-Q is available [24]. To assess LOC eating, the ChEDE-Q uses a two-step process: it first asks whether an unusually large amount of food was consumed. Subsequently, a separate item specifically inquires whether a loss of control was experienced during these episodes. The latter item was used to identify LOC eating.

#### Parent version of the eating disorder examination-questionnaire adapted for children

The ChEDE-QP was developed by the senior author based on the ChEDE-Q as a parent-report version of children’s ED psychopathology and symptoms. Each item was slightly rephrased to address parents instead of children (e.g., “On how many of the past 28 days has your child been scared that it might gain weight?” instead of “On how many of the past 28 days have you been scared that you might gain weight?”). The structure and the rating scheme correspond to that of the ChEDE-Q. Like the ChEDE-Q, the ChEDE-QP consists of 28 items, including 6 diagnostic items and 22 scale-building items. Consistently, Item 14, which addresses whether a loss of control was experienced when eating an unusually large amount of food, was used to assess LOC eating via self-report.

#### Depression inventory for children and adolescents

The German version of the Children’s Depression Inventory [29], DIKJ; [44] was used to assess depressive symptoms in youth aged 8–16 years. The inventory consists of 26 items, each rated on a 4-point Likert scale (0 = *never* to 3 = *very often*). A total sum score is calculated, with higher scores indicating more severe depressive symptoms. The DIKJ was provided with population norms, and the psychometric quality was documented [15].

#### Body mass index (BMI)

Children’s BMI was derived from objectively measured body height and weight. Age- and sex-specific BMI percentiles were calculated based on German reference data by Kromeyer-Hauschild et al. [31]. BMI-Standard Deviation Scores (BMI-SDS) were used to classify children’s weight status into normal weight (− 1.28 < BMI-SDS < 1.28), overweight (1.28 ≤ BMI-SDS < 1.88), and obesity (BMI-SDS ≥ 1.88).

#### Sociodemographic variables

Children’s age, sex (male, female), and education (school type and grade) were assessed by parent report. Participating parents also provided information on their own age, sex (male, female), and socioeconomic status of the participating parent, defined as the highest degree of education.

### Data analytic plan

In order to investigate the ChEDE-QP’s psychometric properties, item distributions were assessed for normality using Shapiro–Wilk and Kolmogorov–Smirnov tests and examining item skewness and kurtosis. Item analysis on the 22 scale-building items included descriptive statistics, the number of missing items, corrected item-total correlations with Pearson`s *r* being interpreted as sufficient if *r* ≥ 0.30 [39], and item difficulty, calculated as *p*m = sum of item scores/(N*maximum item score), with scores closer to 1 indicating easier items [37]. Reliability was evaluated through internal consistency, using Cronbach’s α and McDonald’s ω. Factorial validity of the ChEDE-QP was examined through Principal Component Analysis (PCA) with Varimax rotation of the scale-building 22 items, extracting factors with eigenvalues > 1.

Regarding discriminant validity, LOC + and LOC- groups were compared using *t*-tests for independent samples, focusing on the ChEDE-QP subscale and global scores.

For evaluating convergent validity, the level of agreement between ChEDE-QP, ChEDE-Q, and ChEDE subscale and global scores was analyzed using Pearson’s *r*. The level of agreement for the presence of LOC eating (yes, no) was tested by Cohen’s κ. Additionally, Pearson’s *r* was used to examine relationships between the global scores of the ChEDE, ChEDE-Q, and ChEDE-QP, as well as depressive symptoms (DIKJ percentile rank).

To determine criterion validity of the ChEDE-QP for the presence of LOC eating as assessed by the ChEDE, a hierarchical logistic regression analysis was conducted in two steps. In the first step, the presence of LOC eating (yes/no) according to the ChEDE-Q was included as independent variable. In the second step, LOC eating (yes/no) according to the ChEDE-QP was entered. Additionally, the criterion validity of the ChEDE-QP for the presence of LOC eating as assessed by the ChEDE was evaluated using the ChEDE-QP as the sole independent variable. Variance explanation for the interview-based presence of LOC eating was expressed as Nagelkerke’s *R*^2^.

All statistical analyses were performed using SPSS Statistics®, and a two-tailed α = 0.05 was applied. Effect size classifications and their references are detailed in a supplementary table [see Supplementary material 2].

## Results

An overview of the socio-demographic and anthropometric characteristics for both the total sample and the diagnostic subgroups is presented in Table 1.

**Table 1:** Socio-demographics and anthropometrics for the total sample and diagnostic subgroups

	Total sample *N* = 115	LOC + *n* = 56	LOC- *n* = 59	Cohen ‘s *d*	*t/*χ^*2*^	*df*	*p*
Children	*M (SD*) or *n* (%)	*M (SD*) or *n* (%)	*M (SD*) or *n* (%)				
Age, years	10.73 (1.48)	10.57 (1.46)	10.88 (1.49)	0.21	1.13	113	0.26
Sex					0.02	1	0.90
Girls	65 (56.50%)	32 (57.14%)	33 (55.93%)				
Boys	50 (43.50%)	24 (42.86%)	26 (44.07%)				
Weight status^a^					0.39	2	0.82
Normal weight	51 (43.35%)	26 (46.43%)	25 (42.37%)				
Overweight	28 (24.35%)	14 (25.00%)	14 (23.73%)				
Obesity	36 (31.30%)	16 (28.57%)	20 (33.90%)				
	*M (SD*)	*M (SD*)	*M (SD*)				
BMI, kg/m^2^	22.93 (5.03)	22.56 (4.91)	23.27 (5.16)	0.14	0.75	113	0.45
BMI-SDS	1.28 (1.06)	1.24 (1.07)	1.33 (1.06)	0.09	0.46	113	0.65
BMI percentile	75.16 (26.29)	73.80 (25.96)	76.45 (26.76)	0.10	0.54	113	0.59
Mental comorbidities					4.15	4	0.29
Anxiety disorder	1 (0.87%)	1 (1.79%)	–				
ADHD	1 (0.87%)	–	1 (1.69%)				
Sleep disorder	2 (1.74%)	2 (3.57%)	–				
No diagnosis	111 (96.52%)	53 (94.64%)	59 (98.31%)				
*Parents*							
Participating parents					3.41	1	0.06
Mothers	105 (92.11%)	48 (87.27%)	57 (96.61%)				
Fathers	9 (7.89%)	7 (12.73%)	2 (3.39%)				
	(*M, SD*)	(*M, SD*)	(*M, SD*)				
BMI, kg/m^2^	27.58 (6.43)	28.30 (6.74)	26.92 (6.11)	0.21	1.13	111	0.26
School education^b^					0.70	1	0.40
low	69 (62.15%)	34 (64.15%)	35 (60.35%)				
high	42 (37.85%)	19 (35.85%)	23 (39.65%)				

Mental comorbidities were assessed through the Children’s Diagnostic Interview for Mental Disorders [41, 42]

ADHD: attention-deficit/hyperactivity disorder; LOC, loss of control; BMI, body mass index (kg/m^2^); BMI-SDS, body mass index-standard deviation score

Due to missing data, values may not sum up to *N* = 115 (100%)

^a^Child weight status classification into normal weight (-1.28 < BMI-SDS < 1.28), overweight (1.28 ≤ BMI-SDS < 1.88), and obesity (BMI-SDS ≥ 1.88) based on reference data [31]

^b^Low 5 no school degree, or degree less than 13 years of school education; high 5 degree with 13 years of school education or university degree

Regarding item characteristics and subscale scores of the ChEDE-QP (Table 2), Shapiro–Wilk and Kolmogorov–Smirnov tests indicated nonnormality for all items (*ps* < 0.05). Most items on the WC and SC subscales exhibited moderate skewness, except for item 6 (SC) and item 8 (WC) being severely skewed, as were all items on the RS and EC subscales. Regarding kurtosis, while some items of the SC and WC subscale showed moderate nonnormality, all other items displayed severe nonnormality in kurtosis. Except for item 5 (WC; *r* = 0.15), corrected item-subscale correlations were sufficient for all items (0.33 ≤ *r* ≤ 0.84). The item difficulty ranged from 0.06 ≤ *pₘ* ≤ 0.28 with a tendency towards higher difficulty. Missing item responses occurred in 0–4.35% of items. Little’s Missing Completely at Random (MCAR) test indicated that missing values were not missing completely at random (*p* < 0.5).

**Table 2:** Item characteristics of the ChEDE-QP based on the ChEDE-Q original 4-factor structure comprising restraint, eating concern, weight concern and shape concern

Item	Original subscale	*M*	*SD*	Skewness	Kurtosis	Item difficulty	Corrected item-subscale correlation	*n*	Missing item responses *n* (%)
1	RS	0.61	1.08	2.38	6.65	.10	.57	114	1 (0.87%)
2	RS	0.06	0.40	8.31	81.59	.02	.39	115	0 (0.00%)
3	RS	0.37	1.06	4.01	18.01	.06	.53	114	1 (0.87%)
4	RS	0.53	1.23	2.72	7.12	.09	.33	115	0 (0.00%)
5	RS	0.03	0.27	7.19	55.17	.02	.15	115	0 (0.00%)
6	SC	0.45	1.32	3.40	10.97	.08	.54	113	2 (1.74%)
7	EC	0.17	0.62	5.46	35.74	.03	.56	114	1 (0.87%)
8	WC	0.10	0.45	5.28	30.47	.03	.57	115	0 (0.00%)
9	EC	0.21	0.72	5.60	38.78	.03	.51	112	3 (2.61%)
10	SC	0.79	1.28	1.98	4.02	.13	.77	113	2 (1.74%)
11	SC	1.44	1.81	1.47	1.19	.24	.79	112	3 (2.61%)
12	WC	1.14	1.82	1.73	1.87	.16	.69	113	2 (1.74%)
13^a^		2.68	4.52					114	1 (0.87%)
14^a^		0.47	2.14					112	3 (2.61%)
15^a^		0.28	0.80					110	5 (4.35%)
16^a^		0.01	0.09					115	0 (0.00%)
17^a^		0.00	0.00					115	0 (0.00%)
18^a^		0.40	2.05					115	0 (0.00%)
19	EC	0.64	1.19	2.61	7.74	.11	.52	112	3 (2.61%)
20	EC	0.48	1.06	3.30	12.50	.08	.80	113	2 (1.74%)
21	EC	0.55	1.38	2.81	7.26	.09	.65	113	2 (1.74%)
22	WC	1.0	1.57	1.73	2.21	.17	.81	114	1 (0.87%)
23	SC	1.06	1.42	1.60	2.21	.18	.66	114	1 (0.87%)
24	WC	0.83	1.52	2.05	3.49	.14	.45	113	2 (1.74%)
25	WC	1.69	1.96	1.05	-0.16	.28	.84	113	2 (1.74%)
26	SC	1.68	1.93	1.03	-0.18	.28	.79	114	1 (0.87%)
27	SC	1.24	1.71	1.33	0.73	.21	.70	113	2 (1.74%)
28	SC	1.47	2.00	1.25	0.19	.24	.69	113	2 (1.74%)

ChEDE-QP, eating disorder examination-questionnaire for children adapted for parents; ChEDE-Q, eating disorder examination-questionnaire adapted for children; RS, restraint; EC, eating concern; WC, weight concern; SC, shape concern

^a^Diagnostic items

All ChEDE-QP subscales showed acceptable to excellent internal consistency with Cronbach`s α between 0.72 ≤ α ≤ 0.91 and McDonald`s ω between 0.81 ≤ ω ≤ 0.92. (Table 3; for RS, McDonald`s ω could not be calculated.)

**Table 3:** Reliability of the ChEDE-QP based on the ChEDE-Q original subscale structure and convergent validity assessed via level of agreement between parent and child reports

	*M (SD)*	Cronbach`s α/ McDonald`s ω	Pearson’s r for subscale and global scores		
			ChEDE-QP and ChEDE	ChEDE-QP and ChEDE-Q	ChEDE-Q and ChEDE
Restraint	**0.32 (0.61)**	.72/ -	.14	.31**	.26*
Eating concern	**0.41 (0.75)**	.79/ .81	.07	.18	.43**
Weight concern	**0.95 (1.18)**	.83/ .89	.38**	.45**	.61**
Shape concern	**1.02 (1.20)**	.91/ .92	.36**	.44**	.64**
Global score	**0.68 (0.82)**	.91/ .92	.30**	.46**	.58**
Cohen’s κ for LOC eating (yes/no)			.09	.02	.44**

N = 113–115

^*^ *p* < .05

^**^ *p* < .01

ChEDE-QP, eating disorder examination-questionnaire adapted for parents; ChEDE-Q, eating disorder examination-questionnaire adapted for children; ChEDE, eating disorder examination for children

For the PCA, the Kaiser–Meyer–Olkin measure indicated sampling adequacy (Measure of Sampling Adequacy = 0.826), and Bartlett’s test of sphericity, χ^2^(231) = 1962.781, *p* < 0.001, indicated satisfactorily large correlations between items. The PCA of the 22 scale-building items yielded a three-factor solution for the total sample (eigenvalues > 1), explaining 65.59% of the variance. After orthogonal Varimax rotation, Factor 1 included all WC and SC items and 3 EC items (15 items total). Factor 2 predominantly included the 5 EC items and factor 3 mainly included 4 RS items (7 items total). The primary loadings ranged from 0.31 to 0.83. However, several items showed substantial cross-loadings (≥ 0.30) (Table 4).

**Table 4:** Principal Component analysis with orthogonal varimax rotation with factor loadings of the ChEDE-QP

	Item	Factor 1	Factor 2	Factor 3
Eigenvalues		10.54	2.32	1.56
Variance %		47.92	10.56	7.11
Original subscale				
RS	1			.75
RS	2			.46
RS	3			.76
RS	4			.84
RS	5		.71	
EC	7		.77	
EC	9	.42	.67	
EC	19		.62	
EC	20	.69	.34	
EC	21	.69	.41	.60
WC	8	.35	.68	
WC	12	.53		.36
WC	22	.75		
WC	24	.71	.39	
WC	25	.85		
SC	6	.31		
SC	10	.56		.40
SC	11	.79		
SC	23	.79		
SC	26	.84		
SC	27	.83		
SC	28	.71	.35	

Note that only factor loadings ≥ .30 are displayed

*n* = 104

ChEDE-QP, eating disorder examination-questionnaire adapted for parents; ChEDE-Q, eating disorder examination-questionnaire adapted for children RS, restraint; EC, eating concern; WC, weight concern; SC, shape concern

Descriptive statistics showed that the presence of LOC eating in the LOC + group as determined through the ChEDE was identified by 11.6% (*n* = 13) of the parents using the ChEDE-QP, whereas 40.9% (*n* = 45) of children self-reported LOC eating in the ChEDE-Q. Regarding discriminant validity, the LOC + and LOC- groups did not significantly differ in the ChEDE-QP subscale and global scores (less than small effects; Table 5). In contrast, the ChEDE-Q subscale and global scores differed significantly between both groups (medium-to-large effects), except for RS, which did not differ significantly (less than small effect). In the ChEDE, significant differences between the LOC + and LOC- groups were observed across all subscales and the global score, small-to-large effects.

**Table 5:** ChEDE-QP, ChEDE-Q, and ChEDE subscale and global scores in children with and without LOC eating

	LOC +		LOC-		Cohen’s* d*	*t*	*df*	*p*
	*M*	*SD*	*M*	*SD*				
*ChEDE-QP*								
Restraint	0.32	0.61	0.32	0.61	0.02	− 0.10	113.00	.992
Eating concern	0.40	0.67	0.42	0.84	0.02	0.11	112.00	.917
Weight concern	1.04	1.14	0.87	1.23	0.15	− 0.77	112.00	.440
Shape concern	1.11	1.12	0.95	1.21	0.13	− 0.72	112.00	.475
Global score	0.72	0.76	0.64	0.87	0.10	− 0.53	112.00	.596
*ChEDE-Q*								
Restraint	0.97	1.06	0.78	1.17	0.17	− 0.91	114.00	.363
Eating concern	1.21	1.27	0.39	0.52	0.86	− 4.61	75.53	< .001
Weight concern	2.09	1.79	1.11	1.18	0.65	− 3.45	94.61	< .001
Shape concern	2.10	1.74	1.24	1.29	0.56	− 3.10	105.00	.003
Global score	1.58	1.32	0.88	0.93	0.62	− 3.34	102.34	.001
*ChEDE*								
Restraint	0.58	0.66	0.27	0.51	0.48	− 2.94	110.92	.004
Eating concern	0.56	0.71	0.09	0.19	0.90	− 4.91	67.65	< .001
Weight concern	1.47	1.13	0.75	0.70	0.77	− 4.22	98.10	< .001
Shape concern	1.52	1.31	0.61	0.72	0.86	− 4.69	91.75	< .001
Global score	1.03	0.82	0.43	0.41	0.93	− 5.08	86.72	< .001

Cohen’s *d* is presented as absolute value

CHEDE-QP, eating disorder examination-questionnaire for children adapted for parents; ChEDE-Q, eating disorder examination-questionnaire adapted for children; ChEDE, eating disorder examination for children

Regarding convergent validity, the ChEDE-QP demonstrated significantly positive correlations with the corresponding subscales of the ChEDE for RS, WC, and SC, as well as the global score (medium effects; Table 3). Significant medium-sized correlations were also observed between the ChEDE-QP and the corresponding subscales of the ChEDE-Q, specifically for WC and SC as well as the global score. The ChEDE and the ChEDE-Q were significantly positively associated in all subscales and the global score (small-to-large effects).

Categorically, the ChEDE-QP showed slight agreement with the ChEDE-Q and ChEDE for the presence of LOC eating (κ = 0.11 and 0.03). In contrast, there was a moderate agreement between ChEDE-Q and ChEDE (κ = 0.44) for the presence of LOC eating.

Significantly positive correlations were found between the ChEDE-QP and child-reported depressive symptoms, *r* = 0.41, *p* < 0.01, medium effect. The ChEDE-Q and ChEDE showed significantly positive correlations with child-reported depressive symptoms as well, *r* = 0.39 and *r* = 0.36, medium effects, *ps* < 0.01.

Regarding criterion validity, the hierarchical logistic regression analysis revealed that the ChEDE-Q global score explained approximately 26.3% of variance in the presence of LOC eating assessed via the ChEDE, χ^2^(1) = 23.45,* p* < 0.001, large effect. Upon adding the ChEDE-QP in the second step, the explained variance did not significantly change, Δχ^2^(1) = 0.00,* p* = 0.953, indicating that the addition of the ChEDE-QP did not significantly improve the prediction of LOC eating status. When included as the sole independent variable, the ChEDE-QP nonsignificantly explained 0.3% of the variance in the presence of LOC eating as assessed by the ChEDE, χ^2^(1) = 0.23, *p* = 0.634, less than small effects.

## Discussion

Despite a need for reliable parent-based assessments on children’s disordered eating behaviors, there is only preliminary evidence on the accuracy of existing parent-report questionnaires. The two existing parent-report questionnaire, based on the adult versions of the EDE-Q [10] and the EDE-QS [49], were evaluated in samples with treatment-seeking children and adolescents with predominately restrictive EDs, leaving questions on the questionnaires’ ability to asses LOC eating. This study is the first to examine the psychometric properties of a parent version of a child-adapted self-report questionnaire from the EDE-Q family, the ChEDE-QP, facilitating comparability among child and parental measures. The aim was to provide a complementary perspective to the established questionnaires (EDE-Q and ChEDE-Q) by developing a reliable parent-report measure that assesses children’s eating behaviors from the parent’s perspective, facilitating comparability across parent and child assessments. For validation, both interview (ChEDE) and questionnaire (ChEDE-Q) data from the child were used. The ChEDE-QP showed acceptable reliability and suitable convergent validity in a sample of 115 children aged 8–13 years with and without LOC eating. Internal consistency of the original subscales was good, but factorial validity was only partially confirmed. Convergent validity evidenced medium-sized level of agreement with the ChEDE-Q, the ChEDE, and child-reported depressive symptoms and indicated its potential utility in both research and clinical practice.

Although the MCAR test suggests that missing data were not completely at random, the proportion of missingness was minimal, ranging from 0 to 4% across individual items. Consequently, any systematic bias introduced by these missing values is unlikely to have significantly impacted the overall findings. The relatively low rate of nonresponses also indicates good comprehensibility of the questionnaire, which aligns with previous findings for the ChEDE-Q. [24]. The highest rate of missing responses was observed for item 15 assessing the frequency of OBEs, indicating that parents may have limited insight into this behavior of their children. Corrected item-subscale correlations were sufficient for all items except for item 5 from the RS subscale (empty stomach) suggesting that this item does not align well with the other items in the RS subscale and may not measure the same construct [37]. One possible explanation is that unlike the other items assessing observable behavior, item 5 addresses a wish that parents can only recognize if articulated by the child. This interpretation is supported by an item mean score of 0.03, indicating that item 5 was rarely endorsed. However, as this item also had a low mean score of 0.48 in the ChEDE-Q, the desire for an empty stomach may not be characteristic of this sample, which includes individuals with overweight, both with and without LOC eating. This finding aligns with research by Goldschmidt et al. [17] on the ChEDE-Q, suggesting that this item may be more relevant in samples with AN or BN. Regarding item difficulty, the ChEDE-QP demonstrated relatively low scores, indicating higher difficulty levels. Similar results were observed for the ChEDE-Q in this study (0.07 ≤ *p*ₘ ≤ 0.37). Given that this study was conducted with a nontreatment-seeking sample, it is reasonable to expect that the items would not receive high scores. Skewness and kurtosis showed positive deviations, indicating that most participants showed low ED psychopathology, while a few scored exceptionally high, reflecting that severely disordered behaviors were less common in this sample.

Overall, when the items were assigned to their original scales the ChEDE-QP demonstrated good reliability, with its internal consistency ranging from acceptable (RS, EC) to excellent (SC). This level of reliability is comparable to the findings in studies of the PEDE-Q [10] and the EDE-QS-P [49]. Consistent with previous research, the original four-factor structure of the EDE-Q could not exactly be replicated (e.g., [33]), potentially due to content heterogeneity and/or developmental effects in younger children, which may influence their understanding or interpretation of certain items. Notwithstanding, overall a meaningful factor structure became apparent. The PCA identified three factors collectively explaining 69.78% of the variance, in line with a study on the ChEDE-Q [24]. The first factor showed substantial overlap between SC, WC, and EC, supporting findings on combining SC and WC into one subscale [18], while the second factor was predominantly characterized by EC items, and the third factor by RS items. Strikingly, good internal consistency was observed when the items were assigned to their original scales, which is why the original factor structure was used for further analyses.

For convergent validity, there was a high level of agreement between parental (ChEDE-QP) and child reports (ChEDE-Q and ChEDE) in children’s ED psychopathology. This result aligns with the findings for the PEDE-Q [10] and the EDE-QS-P [49], for which agreement with child self-report (EDE-Q) was found, without application of the (Ch)EDE. Given the medium-sized associations in the global score between the ChEDE-QP and the ChEDE, parent reports may be suitable for providing information on behaviors and attitudes associated with EDs. As to be expected due to the same source of information, associations in global ED psychopathology were larger between ChEDE and ChEDE-Q, in line with previous evidence [4]

Regarding associations of the ChEDE-QP global score and child-reported depressive symptoms, significant medium-sized correlations were found, corresponding to the established link between ED behaviors and distress, consistent with previous research on the ChEDE-Q [24]. However, regarding the presence of LOC eating, the ChEDE-QP only showed slight agreement with both the ChEDE-Q and the ChEDE, while the latter instruments revealed moderate agreement. Specifically, only 11.6% of parents correctly reported the presence of LOC eating in their children, as determined through the ChEDE. However, among children less than half (40.9%) reported LOC eating via the ChEDE-Q, indicating that the underestimation of LOC eating observed in both the children’s and parents’ questionnaires further underlines that the interview is more suitable for capturing children’s LOC eating [11]. This underestimation in children’s self-report might result from the subjective nature of LOC eating or their young age, as they may need assistance in understanding the concept [6]. Thus, parents may not be fully aware of their children’s LOC eating, as demonstrated in previous studies [36, 45], which may be related to the secretive nature of LOC eating or socially desirable responding (to have a healthy child). Despite living in the same household, children might experience LOC eating at school, while away from home, or deliberately hide it from their parents [46]. The discrepancy in parent–child report highlights the challenge in relying on parental reports only for identifying LOC eating in children. A multi-informant approach, as suggested by Kraemer et al. [30], including a perspective from the teacher, grandparents, or friends, in addition to child and parent, could provide valuable insights into the nonhome context.

In terms of the ChEDE-QP’s ability to discriminate between children with and without LOC eating, only small-sized differences were found for the global score indicating weak differentiation, but in line with a low parent–child agreement on LOC eating episodes. In contrast, the LOC + and LOC- groups differed in the global scores from the ChEDE and ChEDE-Q as previously found [24], supporting the assumption of higher convergence of assessments from the same source and parental nonawareness of children’s eating disturbances in a nontreatment seeking sample. Considering that this sample was not treatment seeking, parents were not necessarily expecting their children to have any eating disturbances unless the eating disturbances were observable or communicated by children. Since the discriminant validity of the PEDE-Q [10] and the EDE-QS-P [49] has not been investigated, this study provides valuable information for further research.

The strengths of this study include the matching of the LOC + and LOC- groups based on socio-demographic variables, reducing confounding factors and strengthening the internal validity of the comparisons, the use of both questionnaire-based and interview-based assessments from the children for validation, and homogeneous recruitment including children from the community, enhancing generalizability of findings to a broader, nonclinical population. Similarly, the psychometric properties of the ChEDE-QP in treatment-seeking samples of children remain to be examined. It might be expected that parents of children in clinical samples would provide higher scores on the ChEDE-QP, thereby enhancing the questionnaire’s discriminant validity. Additionally, as the study included children with LOC eating only, no information on the ChEDE-QP’s psychometric properties in children with EDs, such AN, BN, and BED were provided. Moreover, due to the relatively small sample size and previous poor model fit reported in the literature (e.g., [33]) it was decided to perform a PCA of the ChEDE-QP to assess the factorial validity instead of a confirmatory or exploratory factor analysis [35]. A meaningful factor structure consisting of three factors became apparent. Although the ChEDE-QP is based on a German translation of the ChEDE-Q, a language-related influence on the factor structure is unlikely because the translation, accurate in both wording and meaning, was verified through a back-translation procedure by a licensed translator [21]. Given the time of study conduct, only binary options for assessing sex were used, while gender identity was not considered. Future studies should adopt broader frameworks aligned with current diversity, equity, and inclusion guidelines. Finally, the study was conducted in a predominantly white, German sample and therefore might need replication in other culturally racially diverse samples or geographic contexts.

## Conclusions

In summary, the results suggest that the ChEDE-QP is a reliable questionnaire with adequate convergent validity for assessing eating disorder psychopathology in children. Future research should incorporate multiple informants, such as grandparents, teachers, or daycare workers, to determine whether their perspectives can provide a broader and more comprehensive understanding of children’s eating behaviors. Including more fathers and examining the impact of the parent’s gender on the accuracy of reports may be valuable to explore [32]. Moreover, larger samples including a broader range of EDs should be utilized for further validation. Clinically, the results suggest that parents are useful for gaining a general picture of children’s ED psychopathology, which might be especially helpful when the children’s report is not available.

## Supplementary Information

Supplementary material 1

Supplementary material 2

## Data Availability

The datasets used and/or analysed during the current study are available from the corresponding author on reasonable request.

## Abbreviations

AN: Anorexia nervosa
APA: American Psychiatric Association
ARFID: Avoidant/restrictive food intake disorder
BED: Binge-eating disorder
BMI: Body mass index
BMI-SDS: Body mass index–standard deviation score
BN: Bulimia nervosa
ChEDE: Eating disorder examination adapted for children
ChEDE-Q: Eating disorder examination-questionnaire adapted for children
ChEDE-QP: Parent version of the eating disorder examination-questionnaire adapted for children
DIKJ: Depression inventory for children and adolescents (German version)
DSM-5: Diagnostic and statistical manual of mental disorders (5th ed.)
EC: Eating concern (subscale)
EDE: Eating disorder examination
EDE-Q: Eating disorder examination-questionnaire
EDE-QS: Eating disorder examination-questionnaire short
EDE-QS-P: Parent version of the eating disorder examination-questionnaire short
ED: Eating disorder
ICD-11: International classification of diseases (11th rev.)
LOC: Loss of control
LOC +: Group with loss of control eating
LOC-: Group without loss of control eating
MCAR: Missing completely at random
OBEs: Objective binge-eating episodes
PCA: Principal Component Analysis
PEDE-Q: Parent EDE-Q
ROC: Receiver operating characteristic
RS: Restraint (subscale)
SBEs: Subjective binge-eating episodes
SC: Shape concern (subscale)
WC: Weight concern (subscale)
