# Correction: Validation of the parent version of the eating disorder examination‑questionnaire adapted for children in parent–child dyads with children with and without loss of control eating

**DOI:** 10.1186/s40337-025-01417-5

**Published:** 2025-09-29

**Authors:** Caroline Lange, Ricarda Schmidt, Anja Hilbert

**Affiliations:** 1 Department of Psychosomatic Medicine and Psychotherapy, Behavioral Medicine Research Unit, Integrated Research and Treatment Center Adiposity-Diseases, Leipzig University Medical Center, Leipzig, Germany; 2 German Center for Child and Adolescent Health (DZKJ), Partner site Leipzig/Dresden, Leipzig, Germany

**Correction: Journal of Eating Disorders (2025) 13:118** 10.1186/s40337-025-01293-z

Following the publication of the original article [1], the authors reported errors in the data of M(SD) in Table 3, due to a data transfer error. In the originally published table, the means and standard deviations reported for the subscales were those of the Eating Disorder Examination‑Questionnaire adapted for Children (ChEDE-Q), rather than the intended values for the Parent Version of the Eating Disorder Examination‑Questionnaire adapted for Children (ChEDE-QP). The mean and standard deviation of the ChEDE-QP global score as well as data on internal consistencies and correlation coefficients were correctly reported.

The incorrect and correct version of the M(SD) is as follows:

**Table:** 

	*Incorrect*	*Correct*
Restraint	0.87 *(1.12)*	**0.32 (0.61)**
Eating concern	0.80 *(1.05)*	**0.41 (0.75)**
Weight concern	1.58 *(1.58)*	**0.95 (1.18)**
Shape concern	1.67 *(1.58)*	**1.02 (1.20)**
Global score	0.68 *(0.82)*	**0.68 (0.82)**

The original article has been updated accordingly.

## References

[1] Lange C, Schmidt R, Hilbert A. Validation of the parent version of the eating disorder examination-questionnaire adapted for children in parent–child dyads with children with and without loss of control eating. J Eat Disord. 2025;13:118. 10.1186/s40337-025-01293-z. 10.1186/s40337-025-01293-z PMC12180226 40537861

