# Supplementary material for: Validation of the parent version of the eating disorder examination-questionnaire adapted for children in parent–child dyads with children with and without loss of control eating
Source: J Eat Disord. 2025 Jun 19;13:118. doi: 10.1186/s40337-025-01293-z (PMC12180226; doi:10.1186/s40337-025-01293-z)
Supplement: Supplementary file 1 — Supplementary material 1 [file 40337_2025_1293_MOESM1_ESM.docx]

**Supplemental material to**

Validation of the Parent Version of the Eating Disorder Examination-Questionnaire Adapted for Children in Parent-Child Dyads with Children with and without Loss of Control Eating

by Caroline Lange, Ricarda Schmidt, PhD, Anja Hilbert, PhD

**Supplemental Flow Chart**

**Flow Chart 1** Overview of Recruitment, Inclusion and Exclusion of the Participants


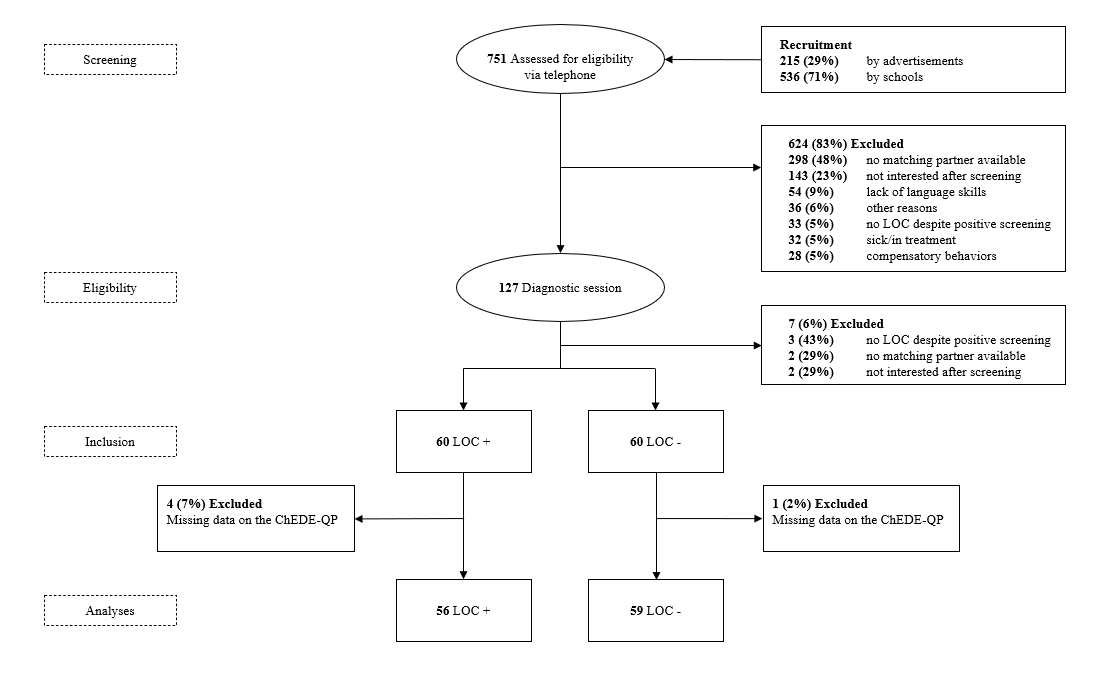


**5, 4.2 %** Excluded

Missing data on the ChEDE-QP

Analyses
