# Supplementary material for: Validation of the parent version of the eating disorder examination-questionnaire adapted for children in parent–child dyads with children with and without loss of control eating
Source: J Eat Disord. 2025 Jun 19;13:118. doi: 10.1186/s40337-025-01293-z (PMC12180226; doi:10.1186/s40337-025-01293-z)
Supplement: Supplementary file 2 — Supplementary material 2 [file 40337_2025_1293_MOESM2_ESM.docx]

**Supplemental material to**

Validation of the Parent Version of the Eating Disorder Examination-Questionnaire Adapted for Children in Parent-Child Dyads with Children with and without Loss of Control Eating

by Caroline Lange, Ricarda Schmidt, PhD, Anja Hilbert, PhD

**Supplemental Tables**

**Supplemental Table 1.** Overview of Effect Sizes, Normality, and Reliability Metrics in Psychometric Analysis

**Supplemental Table 1.** Overview of Effect Sizes, Normality, and Reliability Metrics in Psychometric Analysis

|  | Interpretation | Cronbach’s α | McDonald’s ω | Pearson’s *r* | Cohen’s *d* | R² | Interpretation | Inter-rater reliability κ | Interpretation | Normality |
| --- | --- | --- | --- | --- | --- | --- | --- | --- | --- | --- |
|  | Acceptable/  small | ≥.70 | <.80 | .10 | .20 | .02 | None | <.01 | Normally distributed | 0.00 |
|  | Good/  medium | ≥.80 | <.90 | .30 | .50 | .13 | Slight | .01–.20 | Slightly nonnormal | <1.00 |
|  | Excellent/  large | ≥.90 | ≥.90 | .50 | .80 | .26 | Fair | .21–.40 | Moderately nonnormal | <2.30 |
|  |  |  |  |  |  |  | Moderate | .41–.60 | Severely nonnormal | >2.30 |
|  |  |  |  |  |  |  | Substantial | .61–.80 |  |  |
|  |  |  |  |  |  |  | Almost Perfect | .81–1.00 |  |  |
| Reference |  | DeVellis & Thorpe (2017) | DeVellis & Thorpe (2017) | Cohen (1988) | Cohen (1988) | Hosmer et al. (2013) |  | Landis & Koch (1977) |  | Lei & Lomax (2005) |

**References**

Cohen, J. (1988). *Statistical power analysis for the behavioral sciences* (2nd ed.). Erlbaum.

DeVellis, R. F., & Thorpe, C. T. (2017). *Scale development: Theory and applications* (5th ed.). Sage Publications.

Hosmer, D. W., Lemeshow, S., & Sturdivant, R. X. (2013). *Applied logistic regression* (3rd ed.). Wiley.

Landis, J. R., & Koch, G. G. (1977). The measurement of observer agreement for categorical data. *Biometrics, 33*(1), 159–174. <https://doi.org/10.2307/2529310>

Lei, P. W., & Lomax, R. G. (2005). The effect of varying degrees of nonnormality in structural equation modeling. *Structural Equation Modeling*, 12(1), 1-27. https://doi.org/10.1207/s15328007sem1201_1
